# 935 Development of Japanese Version of LIBRE Profile-SF, a Social Participation Measure for Burn Survivors

**DOI:** 10.1093/jbcr/iraf019.466

**Published:** 2025-04-01

**Authors:** Yukio Sato, Lewis Kazis, Shoko Asakawa, Ryo Yamamoto, Takayuki Shibusawa, Tomohiro Kurihara, Noriko Yamazaki, Junichi Sasaki

**Affiliations:** University of California Keck School of Medicine; Boston University; School of Nursing, The Jikei University School of Medicine; University of California Keck School of Medicine; National Hospital Organization Kumamoto Medical Center; National Hospital Organization Tokyo Medical Center; University of California Keck School of Medicine; University of California Keck School of Medicine

## Abstract

**Introduction:**

Patient-reported outcome measures for burn survivors have primarily focused on physical and psychological aspects, with few tools assessing social participation. This study developed a Japanese version of the Life Impact Burn Recovery Evaluation Profile Short Form (LIBRE Profile-SF) to measure social participation among burn survivors in Japan.

**Methods:**

The translation followed the Principles of Good Practice for the Translation and Cultural Adaptation Process for Patient-Reported Outcomes Measures. Two native Japanese speakers independently conducted forward translations into Japanese, which were then compared and merged into a single forward version. Backward translation was independently done by two Japanese researchers, one of whom was a second-language English speaker. The original source and the backward translation were then compared by the instrument developer and translators, and discrepancies were discussed. After the draft of Japanese version was developed, cognitive debriefing interviews were conducted with 10 burn survivors. Participants were aged 18 years or older, resided in Japan, could understand Japanese, and had sustained burns covering 5%–20% of total body surface area or affecting the face, hands, feet, or perineum. Interviews were conducted face-to-face or via telephone.

**Results:**

Several points were discussed during the Japanese translation process. The English word “comfortable” sometimes means “not bothered” in Japanese. Additionally, the phrases “be limited” and “cannot be” often share similar expressions in Japanese. Cognitive debriefing interviews were conducted, with 60% female participants and 60% of the participants being 65 years or older. Face-to-face interviews accounted for 70% of the total. Feedback highlighted difficulties in understanding double negatives and literal translations. However, these were not revised, as they were understandable and appropriate. After correcting typographical and grammatical errors, the finalized Japanese version of the LIBRE Profile-SF was completed.

**Conclusions:**

Participants reported difficulties understanding the text, despite the translation aiming preserve original meaning. Adaptations of the LIBRE Profile-SF from other countries also noted the unfamiliarity with double negative expressions and time required to comprehend them. Thus, language differences can significantly impact the interpretation of questionnaires.

**Applicability of Research to Practice:**

The reliability and validity of this questionnaire will be evaluated with 100 participants. This study aims to support the clinical application of the Japanese version of the LIBRE Profile-SF, facilitating better patient reintegration.

**Funding for the Study:**

N/A

